# Sleep Apnea Syndrome after Posterior Fossa Surgery: A Case of Acquired Ondine's Curse

**Published:** 2015-01

**Authors:** Elnaz Faraji rad, Mohammad Faraji rad, Shahram Amini, Reza Zare

**Affiliations:** 1 *Department of Neurosurgery, Mashhad University of Medical Sciences, Mashhad, Iran.*; 2 *Department of Anesthesiology, Mashhad University of Medical Sciences, Mashhad, Iran.*

**Keywords:** Central hypoventilation syndrome, Ondine’s Curse, Posterior fossa surgery

## Abstract

**Introduction:**

Ondine’s Curse is a catastrophic but rare condition in adults. It is referred to as a congenital or acquired condition, in which the patient cannot breathe automatically while asleep. Acquired causes of this disease can be any cause affecting the ventrolateral part of the medulla, which is considered to be the breathing center in humans.

**Case Report:**

A 51-year-old woman, with ataxia and the symptoms and signs of rising Intra-Cranial Pressure, who underwent ventriculoperitoneal shunting and removal of tumour, developed episodic apnea during sleep after surgery and hypercapnia when awake. In her post-operative CT scan, some fine spots of hypodensity in the left lateral part of the medulla were observed. She was managed pharmacologically and underwent tracheotomy. After 50 days, she was discharged from the hospital when she was able to breathe normally.

**Conclusion:**

Having experience with this condition after resection of a fourth ventricle tumor, it was found that Ondine’s Curse can be considered as one of the complications of posterior fossa surgery and is curable by proper management.

## Introduction

Ondine's Curse (or Undine's Curse), also called congenital central hypoventilation syndrome (CCHS) or primary alveolar hypoventilation, is a respiratory disorder that is fatal if untreated. Persons afflicted with Ondine's curse classically suffer from respiratory arrest during sleep (1).

In the 1950s, a syndromic disease was first described as long periods of non-breathing during sleep in a patient with bulbar poliomyelitis (2). Then, in 1962, Severinghaus and Mitchell named this disease “Ondine`s Curse” for the first time (2,3). This word comes from an ancient European legend, dating since Paracelsus (1493-1541), in which a Mermaid curses her unfaithful lover to lose all spontaneous body functions including breathing and in turn only makes these functions available on command (2-5).

In 2006, there were only about 200 known cases of Ondine’s Curse worldwide. In all cases, episodes of apnea occur in sleep; but in a few patients, at the most severe end of the spectrum, apnea also occurs while awake (6).Typically, this lesion causes a selective interruption of the descending anterolateral medullocervical pathway, which is responsible for automatic breathing (6,7).

## Case Report

A 51-year-old woman with a history of depression for 2 years and recent morning headaches, nausea, and vomiting, and ataxic gait was admitted in the clinic. During the clinical examination a bilateral papilledema with abnormal cerebellar tests was observed.

**Fig 1:**
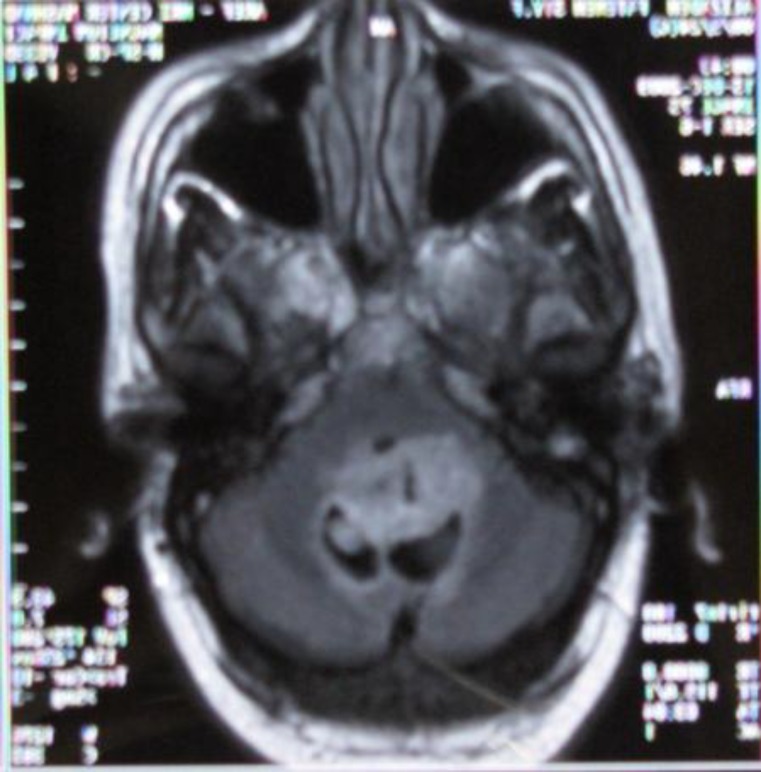
axial FLAIR MRI of the patient shows slightly hyperintense mass lesion in 4^th^ Ventricle).

**Fig 2:**
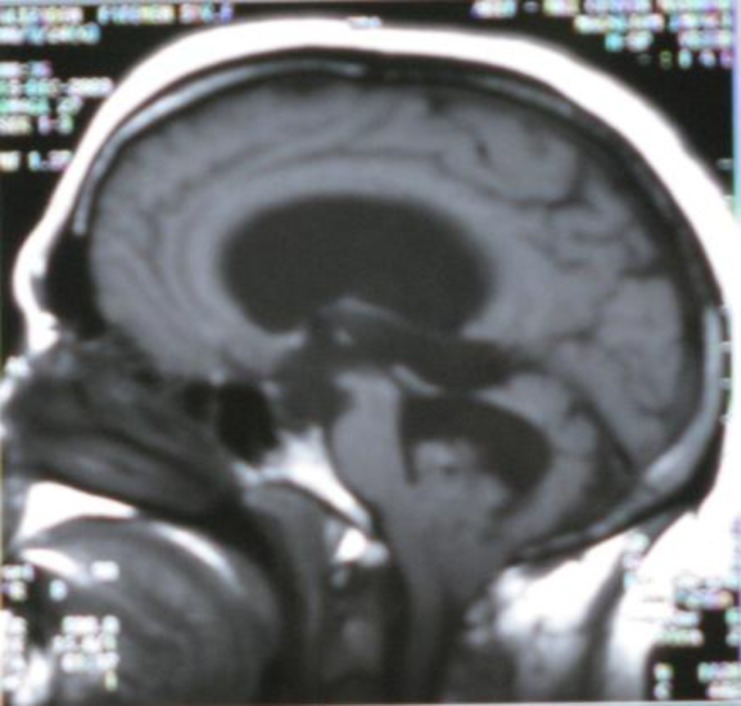
(Sagittal T1-weighted MRI shows isointense tumor in 4^th^ ventricle).

The patient was initially evaluated with a non-contrast CT scan, and it showed three-ventricle hydrocephalus with periventricular edema and a suspicious isodense mass lesion in the posterior fossa. Consequently, an MRI was performed for the patient and it revealed a mass lesion in the fourth ventricle, which was isointense to slightly hyperintense in T1-weighted MRI and iso- to hyperintense in T2-weighted MRI. It appeared that the mass lesion had an attachment to the lateral walls and the floor of the fourth ventricle (Figs.1,2).

Three days after admission, she developed a sudden loss of consciousness, and a ventriculoperitoneal shunting was performed for the patient in an emergent fashion. On the first post operation day, the level of consciousness returned to normal (GCS=15), and an elective surgery for the fourth ventricle lesion was considered.

With no surgical complication, the posterior fossa tumor was resected totally by microsurgery. 

The pathological report of the lesion was compatible with the diagnosis of pilocytic astrocytoma.

The patient was taken to Neuro-ICU and extubated with normal breathing. The night after the operation, the patient developed apneic episodes and decreased O2 saturation, which was displayed by pulse-oxymetry monitoring and confirmed by ABG analysis. So, the patient was urgently incubated and placed on mechanical ventilation (SIMV mode). Interestingly, when the patient was awake, she was able to breathe spontaneously, but during sleep episodes, she was unable to breathe spontaneously and developed apnea.

So, according to the breathing pattern and ABG findings, which showed PCO2=50-60 during apneic episodes and a PCO2 of about 32 mmgh when awake, Ondine’s Curse was the suspected diagnosis. By repeating the ABG test, the breathing pattern was found to be compatible with Ondine’s Curse (Table.1).

**Table 1:** First and second Arterial Blood Gas analysis of the patient during sleeping and awakeness

	**ABG during sleeping**	**ABG during awakeness**
First ABG	Po2=55, Pco2=50	Po2=65,Pco2=35
Second ABG	Po2=50,Pco2=55	Po2=70,Pco2=32

It should be mentioned that all of the cardiopulmonary workup, including chest X-ray and physical examination, was normal during evaluation and the patient did not have any signs or symptoms of pneumonia or fever. 250 mg of Acetazolamide and 10 mg of Medroxyprogestrone was administered four times a day, and a few days later, tracheostomy was performed on the patient.

It should be added that during the post-operative period, the control CT scan revealed hypodense fine spots in the left lateral part of the medulla.

Finally, the patient was discharged from the hospital after 50 days, while she used to have spontaneous breathing while sleeping and awake. After 3 days she was admitted again in the hospital with signs and symptoms of aspiration pneumonia, perhaps due to poor nursing care at home. One month after treatment, she was discharged without tracheostomy and without any other complication.

## Discussion

Almost all published articles in the literature confirm that Ondine’s Curse is a catastrophic but rare disorder in adults. Most of the reported cases of Ondine’s Curse have congenital rather than acquired etiologies, but there are also some reports about several other causes, which can lead to this fatal condition, such as infectious, inflammatory, traumatic, and ischemic events along with vascular malformations and some mitochondrial and demyelinating diseases (e.g Multiple Sclerosis). These involve the brain stem, especially the descending anterolateral medullocervical pathway, which is in charge of spontaneous breathing in humans (1,3,6,7,9,10). 

The suggested criteria for Ondine’s Curse include: hypercapnia during non-REM sleep; hypoventilation during sleep episodes; normal PO2 ABG tests when the patient breathes voluntarily and of course exclude other pulmonary diseases and situations which can mimic this disease (1,11,12).

Genetically, in 2009, Lee et al from National Taiwan University mentioned that the congenital form of Ondine’s Curse - Congenital Central Hypoventilation Syndrome (CCHS) - is due to a mutation in the PHOX2B gene. They found that a family, which used to suffer from CCHS, had hypercapnia and alveolar hypoxia during sleep episodes and more importantly used to have polycythemia with a hematocrit of about 70%. Also, some authors attribute this disease to mal-development of the neural crest, while this disease has also been related to the mutation in the RET-GDNF signaling pathway (2,10,12).

The posterior fossa is a small cavity, which contains vital structures like cerebellum, brainstem, pons, and medulla. Neurosurgical procedures in this area are more challenging than in other parts of the brain.

In the fourth ventricle tumours, the most difficult part of tumour resection is detaching the tumour from the floor of the fourth ventricle; and sometimes it is impossible. Fortunately, in this case the tumour was resected from the floor of the fourth ventricle, which contains important cranial nerve nuclei. One of these important nuclei, which can be damaged, is the breathing drive center that is located in the ventrolateral part of the medulla. Any inflammatory, ischemic, neoplastic, traumatic, and infectious damage can produce significant complications such as Ondine’s Curse.

In Ondine’s curse, as stated before, the patient cannot breathe automatically, and breathing must be done on command. However, during sleep, when the patient is not awake the respiratory drive cannot work spontaneously and this causes hypoventilation and apnea (1,12).

To confirm Ondine’s Curse, some criteria like hypercapnia and hypoventilation during deep sleep (NON-REM SLEEPING which happens in the first hour of sleeping), normal PO2 in ABG tests when the patient is awake, and voluntary breathing are needed (13). But, as mentioned before, there is no absolute agreement on these criteria, and in fact only one criteria may be necessary: the proof that the patient cannot breath automatically but can breathe on command may be enough to diagnose this disease.

Practically, although different therapeutic methods have been proposed for the treatment of Ondine’s Curse, like Phrenic Nerve Stimulation (10,14-16) and some pharmacologic agents, which induce metabolic acidosis (e.g.Acetazolamide), the main goal in the management of this fatal situation is still intensive care of patient’s airway by tracheostomy and mechanical ventilation. In the literature, different outcomes and prognoses have been reported for Ondine’s curse, and the real outcome and prognosis of this disease is unclear and still remains to be further evaluated.

According to this paper, the most important aspect of this disease is proper management of the patient with intensive respiratory support or by some intervention like a diaphragm pace maker. Depending on the etiology, the prognosis can vary between patients.

In this patient, according to the location of the tumour and despite the minimal use of bipolar coagulation, sacrificing some capillaries and venules was inevitable. It is thought that this could be the cause of the brainstem damage in the left part of the medulla, which can lead to developing Ondine’s Curse in the patient.

So, according to this case, Ondine’s Curse can be considered as one of the most important complication of posterior fossa surgery, which can be fatal, unless managed properly.

However, in this case because of our early suspicion to this disease and immediate management of this harsh condition medically and by performing tracheostomy, the patient could be managed successfully and was able to compel the remaining breathing centers to compensate for the lost ones.

## Conclusion

Ondine’s curse is known as a congenital disorder, which is mostly seen in children and is rare in adults. In adults, it is almost always due to some acquired cause, such as neoplastic, inflammatory, traumatic, ischemic, surgical, or mitochondrial diseases. The exact criteria for the diagnosis of this disease are not clear, and also the prognosis is variable according to pathology and the method of management, but the main goal of treatment is respiratory support. This fatal disease should be considered after posterior fossa surgery in patients with abnormal breathing or in patients who are dependent on mechanical ventilation for a long time. Being familiar with Ondine’s Curse can help early diagnosis and allows for the consideration of more serious methods of treatment.
